# Cultivation of stable, reproducible microbial communities from different fecal donors using minibioreactor arrays (MBRAs)

**DOI:** 10.1186/s40168-015-0106-5

**Published:** 2015-09-30

**Authors:** Jennifer M. Auchtung, Catherine D. Robinson, Robert A. Britton

**Affiliations:** Center for Metagenomics and Microbiome Research, Department of Molecular Virology and Microbiology, Baylor College of Medicine, One Baylor Plaza, Houston, TX 77030 USA; Department of Microbiology and Molecular Genetics, Michigan State University, 567 Wilson Rd, East Lansing, MI 48823 USA; Present address: Institute for Molecular Biology, University of Oregon, 249 Klamath, Eugene, OR 97403 USA

**Keywords:** Human microbiome, Cultivation, Bioreactors, MBRA, Microbial communities

## Abstract

**Background:**

Continuous-flow culture models are one tool for studying complex interactions between members of human fecal microbiotas because they allow studies to be completed during an extended period of time under conditions where pH, nutrient availability, and washout of waste products and dead cells can be controlled. Because many of the existing well-validated continuous-flow models are large and complex, we were interested in developing a simpler continuous-flow system that would allow microbial community dynamics to be examined in higher throughput while still maintaining complex microbial communities. To this end, we developed minibioreactor arrays (MBRAs), small volume bioreactors (15 ml) that allow simultaneous cultivation of up to 48 microbial communities in a single anaerobic chamber.

**Results:**

We used MBRA to characterize the microbial community dynamics of replicate reactors inoculated from three different human fecal donors and reactors seeded with feces pooled from these three donors. We found that MBRA could be used to efficiently cultivate complex microbial communities that were a subset of the initial fecal inoculum (15–25 % of fecal OTUs initially observed). After an initial acclimation period of approximately 1 week, communities in each reactor stabilized and exhibited day-to-day variation similar to that observed in stable mouse fecal communities. Replicate reactors were predominately populated by shared core microbial communities; variation between replicate reactors was primarily driven by shifts in abundance of shared operational taxonomic units (OTUs). Consistent with differences between fecal donors, MBRA communities present in reactors seeded with different fecal samples had distinct composition and structure.

**Conclusions:**

From these analyses, we conclude that MBRAs can be used to cultivate communities that recapitulate key features of human fecal communities and are a useful tool to facilitate higher-throughput studies of the dynamics of these communities.

**Electronic supplementary material:**

The online version of this article (doi:10.1186/s40168-015-0106-5) contains supplementary material, which is available to authorized users.

## Background

The gastrointestinal microbiome plays an important role in health and disease (reviewed in [1–8]). Although many insights about the role of the microbiota have been gained by studying microbial community association with the human host (e.g., [9–11]), the availability of less complex models of the microbiota (i.e., conventional and humanized animal ([12–16] and in vitro models [17–22]) have also played an important role in elucidating the roles of the microbiota.

Continuous-flow culture models are beneficial for studying the complex interactions between members of the host microbiota in vitro because they allow for studies to be completed during an extended period of time under conditions where pH, nutrient availability, and washout of waste products and dead cells can be better controlled (reviewed in [23–25]). Although there are several well-studied and validated in vitro models of human microbiota (e.g., Simulator of Human Intestinal Microbial Ecosystem (SHIME, [21, 26, 27]), the TNO gastrointestinal model (TIM-2, [19, 28]) and the three-stage compound continuous culture system [18, 20]), we were interested in developing a simpler, higher-throughput continuous culture system for human fecal communities.

To this end, we developed minibioreactor arrays (MBRAs, [29]). MBRAs were strips of six replicate bioreactors (each with a 15-ml operating volume) that were positioned on a 60-position magnetic stir plate. Continuous-flow was controlled by two 24-channel peristaltic pumps. Because of their relatively small size and simplistic design, up to 48 reactors could be run simultaneously in a single anaerobic chamber, thereby reducing the time and cost needed to evaluate multiple experimental perturbations to microbial communities.

Previously, we demonstrated that fecal microbial communities established in our MBRAs recapitulate one important aspect of healthy microbial communities—the ability to resist colonization by *Clostridium difficile* unless perturbed by antibiotics [29]. Further, these MBRA communities revealed differences in physiology between *C. difficile* epidemic strains that were supported by experiments in a humanized microbiota mouse model of *C. difficile*, providing additional support for the applicability of this model [29].

These initial studies primarily focused on *C. difficile* physiology in the context of disrupted MBRA communities and did not more broadly characterize the types of communities that could be cultivated. A more thorough characterization of the types of communities that could be cultivated in unperturbed MBRA was needed to evaluate the suitability of this platform for other studies characterizing microbial community dynamics and function. Therefore, we followed changes in microbial community structure over time in replicate MBRAs inoculated from three different fecal donors as well as MBRAs inoculated from a pool composed of these three donors.

We found distinct communities could be cultivated from each donor. Community composition stabilized within ~7 days of flow (~21 reactor turnovers based upon the 8-h retention time of MBRAs) to contain a core set of 40–45 operational taxonomic units (OTUs; clustered with ≥97 % ANI across the V4 region of the 16S rRNA gene) across replicate reactors from the same donor. These core set of OTUs contained ~65–95 % of the total sequences. Cultivation resulted in restructuring of the starting fecal communities, with modest decreases in Firmicutes, Actinobacteria, and unclassified bacteria coupled to increases in Bacteroides, Proteobacteria, and Verrucomicrobia. From our studies, we conclude that communities cultivated in MBRAs recapitulate key features of human fecal microbiota and that MBRAs are a useful tool to facilitate higher-throughput studies of the dynamics of these communities.

## Results and discussion

### Diverse microbial communities can be cultivated in MBRA

We inoculated triplicate reactors with fecal samples from one of three healthy donors (donor A, donor B, or donor C) or six replicate reactors with an equal mass of fecal sample pooled from each of the three donors (pool). After an initial acclimation period (16 h), we collected samples (day 1) and initiated continuous flow operation of the reactors with an 8-h retention time. We then collected samples from all communities daily for 20 additional days (days 2–21). We monitored changes in microbial communities by amplifying and sequencing the V4 region of the 16S rRNA gene from these samples as well as from the initial fecal inocula (day 0). We then quality-filtered the data and clustered the sequences into OTUs with ≥97 % average nucleotide identities before further analyses.

**Fig. 1:**
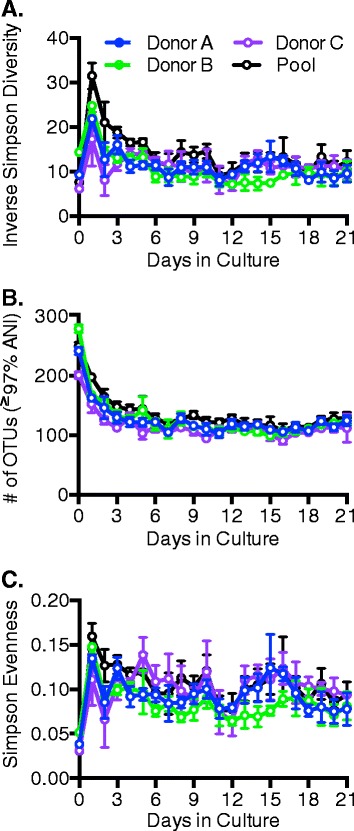
Impact of MBRA cultivation on microbial diversity. Microbial diversity of triplicate MBRA communities inoculated with one of three donor fecal samples (Donor A, *blue circles*; Donor B, *green circles*; Donor C, *purple circles*) or in six replicate MBRA communities inoculated with an equal mass of all three donor fecal samples (Pool, *black circles*) was determined by sequencing the V4 region of the 16S rRNA gene from samples collected daily over 21 days in culture. Microbial diversity (Inverse Simpson, **a**), total number of OTUs (**b**), and evenness of OTU distribution (Simpson Evenness, **c**) was calculated from OTUs (≥97 % average nucleotide identity (ANI)) that were randomly subsampled to 10,000 sequences over 100 iterations. The mean value for the replicate reactors as a function of time in culture is plotted (day 0 = fecal inoculum; *error bars* represent standard deviation of the mean)

We found that cultivation in the MBRAs supported growth of diverse microbial communities. Microbial diversity, as measured by either the Inverse Simpson Index (Fig. 1a) or Shannon Index (see Additional file 1A for graph), was similar between the starting fecal inocula and the MBRA communities on days 2–21 in culture. Cultivation resulted in a decrease of the overall number of OTUs by approximately twofold relative to the starting fecal inocula (Fig. 1b); this was primarily due to loss of low abundance OTUs (OTUs with one sequence in the fecal samples, see Additional file 1B for graph). Consistent with a decrease in the number of low abundance OTUs, the overall evenness of OTU distribution increased approximately two- to threefold in the MBRA communities relative to the starting fecal inocula (Fig. 1c).

We observed that both microbial diversity and evenness spiked on day 1, after 16 h of incubation in medium and prior to the initiation of flow. This transient burst in diversity and evenness likely reflects the unique nature of the sample, which contained those community members that had begun growing as well as those community members that will not grow, either because they were non-viable or could not be cultivated under these conditions. (The method that we used to measure community composition, amplification of the 16S rRNA gene, does not distinguish live from dead cells.) However, once flow was initiated, non-viable and non-growing strains were lost by dilution and turnover within the MBRA, and the remaining community members were those capable of growth in the distinct MBRA communities.

### MBRA cultivation impacts community composition and structure

**Fig. 2:**
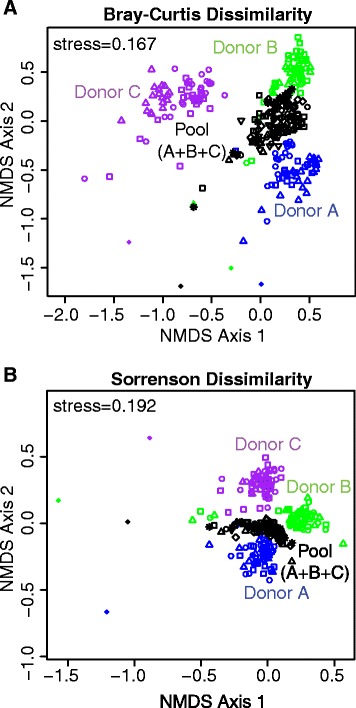
Impact of MBRA cultivation on community composition and structure. Using the data described in Figure 1, we determined pairwise relationships between samples from MBRA communities inoculated with different fecal samples and their respective fecal inocula using (**a**) Bray-Curtis and (**b**) Sorenson dissimilarity measures and plotted this data with non-metric multi-dimensional scaling. Fecal samples = solid diamonds; MBRA communities = *open symbols*, with replicate 1 = *squares*, replicate 2 = *circles*, replicate 3 = *triangles*, replicate 4 = *diamond*, replicate 5 = *inverted triangle*, replicate 6 = *asterisks*; donors A, B, C, and the pool = *blue*, *green*, *purple*, and *black*, respectively. The stress for each NMDS plot is indicated

We next examined the impact of cultivation on the composition and structure of MBRA microbial communities. We determined the relationships between communities present in each sample using two similarity measures, Bray-Curtis similarity (Fig. 2a; Bray-Curtis similarity = 1-Bray-Curtis Dissimilarity), which calculates community similarity as the ratio of sequences in shared OTUs to total sequences, and Sorenson similarity (Fig. 2b; Sorenson similarity = 1-Sorenson Dissimilarity), which calculates community similarity as the ratio of shared to total OTUs [30]. We visualized the relationships between samples with non-metric multi-dimensional scaling (NMDS). As anticipated from previous studies (e.g., [31]), each individual fecal community was distinct. Although cultivation resulted in shifts in microbial composition compared to the starting fecal inocula (discussed more below), MBRA communities from each fecal donor (A, B, or C) rapidly formed distinct communities (Fig. 2a, b), with significant differences between communities inoculated from different fecal donors present by day 2 in culture.

The significance of differences detected between communities on days 2–21 were evaluated with two non-parammetric tests of community similarity, analysis of similarities (ANOSIM) [32] and PERMANOVA [33]. ANOSIM compares the rank similarities of samples within and between groups, generating an R-statistic varying from 1 (rank similarities within all replicates of a group are more similar than rank similarities between groups) to 0 (replicates within and between groups have similar rank similarities). Statistical significance is determined by comparing the R-statistic to the null distribution of R, which is calculated from iterations of randomly permuted data. PERMANOVA evaluates difference in community composition by calculating the differences within and between groups based upon the sum of squared distances from the centroid. The magnitude of the differences within and between groups is reflected in the pseudo-*F* value (*F* value >>1, reflects increasing differences in community composition), and the significance of this value is determined by random permutation. Using both tests for differences in community distributions, we observed that communities were separated with high significance (*p* = 0.001 based upon 999 permutations) and that this separation was typically large (ANOSIM *R* values >0.89 and PERMANOVA F-statistics >55, Table 1).

**Table 1:** Evaluation of variation between MBRA communities inoculated with different fecal samples by analysis of similarities (ANOSIM, [32]) and permutational multivariate analysis of variance (PERMANOVA, [33])

	Bray-Curtis similarity			Sorenson similarity		
	ANOSIM R-statistic	PERMANOVA		ANOSIM R-statistic	PERMANOVA	
		*F* value	*R* ^2^		*F* value	*R* ^2^
Donor A-donor B	0.94	89.5	0.44	0.9	54.8	0.32
Donor A-donor C	0.99	106.7	0.49	0.98	76.4	0.4
Donor B-donor C	0.99	115.0	0.5	0.93	61.6	0.35
Donor A-pool	0.58	40.9	0.19	0.65	35.1	0.17
Donor B-pool	0.57	48.8	0.21	0.74	39.2	0.18
Donor C-pool	0.97	106.9	0.38	0.9	68.8	0.28

All *p* values were 0.001 based upon 999 permutations

MBRA communities inoculated with the pooled samples formed distinct communities. Comparison of differences between the communities formed from the pooled fecal sample to communities formed from donor A, B, or C also demonstrated that the donor C communities were less similar to the pooled communities than either donor A or donor B communities. These data indicate that the a larger proportion of community members present in donor C may not be able to compete with community members present in donor A or B during cultivation in MBRA.

### MBRA cultivation leads to stable microbial communities

**Fig. 3:**
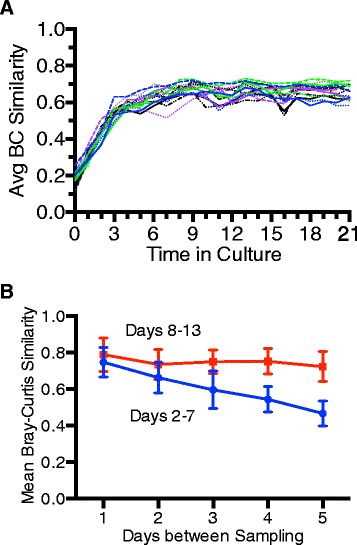
Stabilization of MBRA microbial communities (**a**). MBRA community stability was assessed by plotting the average Bray-Curtis (BC) similarity between each daily bioreactor sample and other days in culture as a function of time in culture. The point at which reactors reached stability was defined as the inflection point of the curve and varied from day 8–12 (median = day 8). **b** The mean Bray-Curtis similarity (± standard deviation) for samples at increasing time intervals were calculated for all reactors over the indicated time intervals (days 2–7 (transitioning communities) and days 8–13 (stable communities)) and plotted as a function of days between samples. Statistical testing of each time interval with an unpaired student’s *t* test demonstrated that the differences in similarity observed between transitioning (days 2–7) and stable (days 8–13) were significant (*p* ≤ 0.03)

Community stability can be challenging to define, as one must decide how much variation is acceptable in a stable community. One previously reported measure for stability [34] that we applied to our data was to plot the mean Bray-Curtis similarity for each day’s sample relative to all other days in culture as a function of time in culture (Fig. 3a). From this plot, we observed that mean similarity values increased rapidly over the first 3 days in culture (slope of line from day 0 to day 3, 0.13 ± 0.04 (mean ± SD)), continued to increase at a reduced rate through day 7 (Slope of line from day 4 to day 7, 0.019 ± 0.01), and plateaued around day 8 in culture (slope of line from day 8 to day 11, −0.009 ± 0.01). More precisely, the inflection point of each curve (i.e., point where slope transitioned from positive to negative) was identified by determining the slopes of each line with three-point sliding windows; the inflection point varied from day 8 to day 12, with a median of 8 and a mean of 8.6. By day 8 in culture, communities had experienced 7 days of continuous flow (~21 reactor turnovers with 8-h retention time). A second measure of stability [35], calculating the similarity between reactors as a function of increasing days in culture, demonstrated similar stabilization dynamics (Fig. 3b).

To better resolve the differences in community structure in stable bioreactor communities, we re-calculated the mean Bray-Curtis dissimilarity for each day’s sample for days 8–21 and plotted this as a function of time (see Additional file 2). We observed that the mean Bray-Curtis similarities from day-to-day ranged from 0.58 to 0.86, with a mean of 0.74 ± 0.05.

Previous studies have shown that variations in sampling, extraction, and sequencing can lead to introduction of artificial variation among samples (e.g., [35, 36]). In order to determine the amount of technical variation present in our data, we performed duplicate sequencing of three identical samples (described in the “Methods” section) and analyzed as described above. We found that the mean Bray-Curtis similarity between these technical replicates was 0.87 ± 0.04 (Additional file 2, red line). Thus, the mean variation in stable reactor communities was twice that observed in technical replicates; this twofold difference correlates to increased variation of sequence distribution of ~13 % in stable reactor communities relative to the controls.

### Variation in microbial communities increases between replicate reactors

Having identified the window in which communities stabilize within each reactor (days 8–21), we next wanted to examine how these communities varied across replicates from the same fecal donor. We determined the mean similarities between replicate reactors inoculated from the same donor on days 8–21 and compared this to the mean similarities observed within each reactor from day-to-day on days 8–21 (Table 2). We observed that Bray-Curtis similarities decreased ~1.7–1.8-fold between replicate reactors. (Mean similarity values ranged from 0.54 ± 0.07 to 0.61 ± 0.08.) This divergence occurred primarily within the first 5 days of cultivation and did not increase significantly once individual reactors had stabilized (see Additional file 3 for graph of similarities between replicate reactors over time). In contrast, Sorenson similarity decreased ~1.1–1.2-fold between replicate reactors (Table 1; mean similarity values from 0.67 ± 0.04 to 0.70 ± 0.04). Although these decreases in Bray-Curtis similarities were modest, these data indicate that changes in the relative abundance of shared OTUs is one potential mechanism that led to differences between replicate reactors.

**Table 2:** Comparison of mean Bray-Curtis and Sorenson similarities for OTUs present in MBRA communities on days 8–21

Reactor type	Within reactor^a^	Between replicates^b^	Between reactor types^c^			
			A	B	C	Pool
Bray-Curtis						
Donor A	0.73 ± 0.08	0.54 ± 0.07	NA	0.29 ± 0.06	0.26 ± 0.05	0.47 ± 0.08
Donor B	0.79 ± 0.07	0.61 ± 0.08	0.29 ± 0.06	NA	0.25 ± 0.06	0.46 ± 0.10
Donor C	0.73 ± 0.10	0.55 ± 0.07	0.26 ± 0.05	0.25 ± 0.06	NA	0.32 ± 0.07
Pool	0.72 ± 0.09	0.57 ± 0.07	0.47 ± 0.08	0.46 ± 0.10	0.32 ± 0.07	NA
Sorenson						
Donor A	0.74 ± 0.05	0.69 ± 0.04	NA	0.55 ± 0.05	0.52 ± 0.05	0.64 ± 0.04
Donor B	0.71 ± 0.05	0.67 ± 0.04	0.55 ± 0.05	NA	0.53 ± 0.05	0.61 ± 0.04
Donor C	0.73 ± 0.04	0.67 ± 0.04	0.52 ± 0.05	0.53 ± 0.05	NA	0.57 ± 0.04
Pool	0.74 ± 0.04	0.7 ± 0.04	0.64 ± 0.04	0.61 ± 0.04	0.57 ± 0.04	NA

^a^Mean ± SD across all replicates of similarities within each replicate reactor of the indicated fecal type

^b^Mean ± SD across all pairwise comparisons of similarities between replicate reactors of the same fecal type on days 8-21

^c^Mean ± SD across all replicates of similarities between reactors of the different fecal types on days 8-21

### Comparison of variation in stable reactor communities to stable mouse communities

To gauge how MBRA community variation compares with another experimental model of gastrointestinal community dynamics, we compared our data to that published by Schloss and colleagues [37], who examined fecal microbial community stabilization in mouse microbial communities post-weaning. In this work, they observed that mouse communities exhibited the greatest variation in community structure in the first 9 days post-weaning and that community structure had stabilized by days 141–150 post-weaning. Therefore, we compared community dynamics in our stable MBRA communities (days 8–21) to differences in community dynamics within each mouse from day-to-day and between different mice over time. Although these models of GI community dynamics have several differences that could impact community dynamics (murine vs human microbiota; presence of host to provide different nutrients, niches, and selection from immune system), we were interested in examining the similarities and differences between community dynamics in these disparate models. We found that community dynamics were quite similar between mice and MBRAs.

Because their original work analyzed stability with a different dissimilarity measure (θ_YC_) and used data generated by pyrosequencing the V3–V5 region of the 16S rRNA gene, we reanalyzed a subset of their data, which was generated by Illumina sequencing of the V4 region and used to cross-validate a new dual-indexing sequencing approach for community analyses [37], using the methods described above. Further, as they observed neither litter, co-housing status, nor sex of the mouse significantly impacted community structure, we selected data from three male and three female mice and treated these mice as independent replicates for our analyses.

#### Day-to-day variation within each mouse

We calculated the mean Bray-Curtis similarity within mice with stable communities to be 0.79 ± 0.06 (see Additional file 4 for a table of mean dissimilarities in stable and unstable mouse communities). Sorenson similarity values were similar, with mean within mouse values of 0.76 ± 0.04. From these data, we conclude that stable individual MBRA communities exhibit similar day-to-day variations as those found in stable murine communities.

#### Variation between replicate mice

The variation in Bray-Curtis similarities between replicate mice with stable communities was 0.71 ± 0.05 (see Additional file 4), which is ~10–15 % lower than the similarity observed between replicate reactors (0.54 ± 0.07 to 0.61 ± 0.08 to, Table 2). In contrast, the Sorenson similarity values between replicate reactors (0.67 ± 0.04 to 0.70 ± 0.04, Table 2) and replicate mice (0.72 ± 0.04, see Additional file 4) were similar.

One potential contributing factor to the higher variation observed in replicate reactors is the higher abundance of Firmicutes in MBRA communities compared to the mouse communities (56 ± 13 % in MBRA; 28 ± 8 % in mice). Flores et al. [38] examined temporal stability in human fecal communities over time and found those subjects that exhibited higher variation in community structure over time also had a higher ratio of Firmicutes/Bacteroidetes than those subjects with lower variation in community structure. As is discussed in more detail below, we found that the distribution of OTUs belonging to the Firmicutes and Proteobacteria phyla were more variable in our samples than OTUs belonging to the Bacteroidetes*.*

Another possible contributing factor to the lower variation observed in mouse data is that communities colonizing these mice are highly adapted to co-existence in the murine GI tract, where selection can be imposed by interactions with host cells, other members of the microbiota, and nutrients from the host diet. In contrast, cultivation in MBRAs could allow organisms with functional redundancy under MBRA cultivation conditions to fluctuate stochastically during stabilization until stable communities are reached.

### Composition of core MBRA communities and comparison to starting fecal inocula

To gain further insights into the composition and structure of MBRA communities, we identified those OTUs that were conserved across samples (i.e., core communities). We identified core communities on three different levels: OTUs found conserved from day-to-day over days 8–21 within each single reactor (individual core), OTUs shared from day-to-day over days 8–21 between replicate reactors of the same fecal donor (fecal type core), and OTUs common to all reactor communities over time (all MBRA core). We also compared the composition of these core communities to their starting fecal inocula.

#### Identification of core communities

**Fig. 4:**
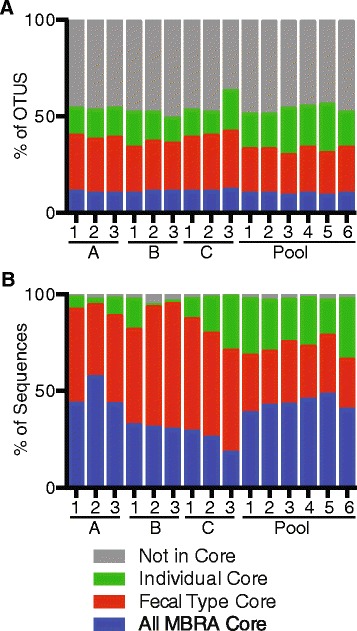
Structure of MBRA core microbial communities. We designated those OTUs that were present in ≥90 % of daily samples over days 8–21 within each single reactor over time as members of the “individual core.” Individual Core OTUs that were shared across replicates of the same fecal type were designated members of the “fecal type core” and those present in the core of all MBRA communities were designated “all MBRA core.” We calculated the mean percent abundance of OTUs (**a**) and sequences (**b**) in each type of core from reactors on days 8–21 and plotted these values for each replicate (1–3 for donors A, B, or C; 1–6 for pooled fecal donor). Each core type includes those members also present in the broader core type (i.e., individual core = individual core + fecal type core + all MBRA core; fecal type core = fecal type core + all MBRA core)

OTUs were designated as members of a core community if they were present in at least 90 % of daily samples collected from a single reactor between days 8–21. Using these criteria, we found that the core communities maintained from day-to-day within each reactor varied from 55 to 72 OTUs (~50–60 % of total OTUs from each day sampled; Fig. 4a (individual cores)). These individual core OTUs contained 95–98 % of the sequences from each day sampled (Fig. 4b). When we determined the overlap between OTUs found in the core communities of replicate reactors of from the same fecal donor, we found that the core community shared between replicate reactors of the same fecal type was composed of 40–45 OTUs (~30–40 % of total OTUs, Fig. 4a (fecal type core)) and contained 66–95 % of sequences present in each reactor from each day sampled (Fig. 4b). Finally, we determined the overlap in core membership across MBRA replicates from all fecal types and found that this all MBRA core contained 12 OTUs comprising 18–48 % of total sequences from each reactor across every day sampled (Fig. 4; also see Additional file 5 for a table listing the 12 OTUs comprising the all MBRA core and their abundances across samples).

#### Comparison of MBRA communities with fecal inocula

We also examined the phylogenetic distribution of OTUs present in the MBRA core communities and compared this with the starting fecal inocula. Because Individual Core OTUs contained 95–98 % of the MBRA sequences, we limited our analyses to these 144 OTUs. In addition, we included those 28 OTUs that were abundant in fecal samples (≥0.5 % of sequences) and were not present in any of the individual core communities (see Additional file 6 for a table listing the 28 OTUs abundant in fecal samples and absent from individual cores their abundances across fecal samples). Limiting our analyses to these 172 OTUs greatly simplified the amount of data while still representing the distribution of ≥94.5 % of the sequences from every sample.

**Fig. 5:**
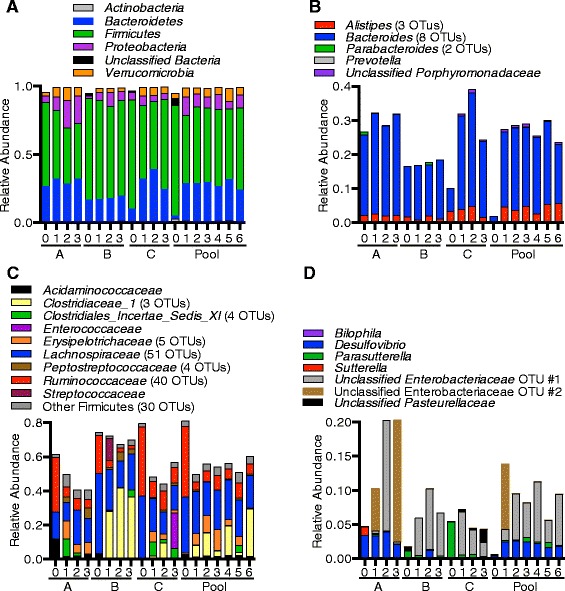
Composition of MBRA core communities and comparison with fecal inocula. We analyzed the phylogenetic distribution of OTUs in the individual core communities and compared this with the phylogenetic distribution of the original fecal inocula. To provide better representation of the fecal inocula, those OTUs absent from the individual core communities that contributed at least 0.5 % of sequences to a fecal sample were also included in our analyses. As in Fig. 4, data present is the mean abundance for each OTU across days 8–21. Following determination of consensus classifications for each OTU (as described in Methods), we plotted the percent abundance of sequences in each phylum (**a**), genus within Bacteroidetes (**b**), family within Firmicutes (**c**), and genus within Proteobacteria (**d**). If a consensus classification for the phylogenetic level plotted could not be determined with confidence, the next highest classification assigned with ≥80 % is given preceded by the designation “unclassified.” To simplify presentation of abundances in **c**, several families with low abundance across all samples were condensed in to the designation “other Firmicutes”, which includes *Clostridiales Incertae Sedis XIII*, *Eubacteriaceae*, *Incertae Sedis XI*, unclassified Firmicutes, unclassified Clostridia, unclassified Clostridiales, and Veillonellaceae

At the phylum level, MBRA communities exhibited similar trends across replicate reactors and community types (Fig. 5a). Sequences classified as Firmicutes were the most abundant members of the community, ranging from 44–70 %, followed by Bacteroidetes (18–32 %), Proteobacteria (5–17 %), and Verrucomicrobia (3–8 %). At this level of analyses, donors A and B MBRA communities were also similar to their starting fecal inocula, whereas donor C and the pool MBRA communities were different. In the fecal inoculum for donor C, the ratio of Firmicutes/Bacteroidetes sequences was higher (80 % Firmicutes sequences/10 % Bacteroides sequences) than the ratio in the fecal inoculum from either donor A (61 % Firmicutes*/*27 % Bacteroidetes sequences) or donor B (fecal inoculum in the fecal inoculum from donor C (74 % Firmicutes/17 % Bacteroidetes). Growth under MBRA cultivation conditions led to a decrease in the ratio of Firmicutes/Bacteroides in reactor communities seeded from donor C (mean ratio in MBRA communities from donor C: 56 % Firmicutes/32 % Bacteroidetes), which was more similar to that observed for MBRA communities seeded with other fecal samples. The phylogenetic distribution of the pooled fecal inoculum was unexpected, with only 2 % of sequences from Bacteroidetes, 81 % of sequences from Firmicutes, 3 % of sequences from Actinobacteria and 4 % of sequences from unclassified bacteria. The levels of Bacteroidetes were 5–14-fold lower than observed in any of the individual fecal samples used to generate the pool; whereas the levels of Actinobacteria and unclassified bacteria were 15–37-fold and 3–15-fold higher, respectively, than any of the individual fecal samples used to generate the pool. (Levels of *Firmicutes* (81 % of sequences), Proteobacteria (2 % of sequences), and Verrucomicrobia (4 % of sequences) were less than twofold different from at least one of the individual inocula.) This distribution could have been caused by heterogeneity in the individual fecal samples used for preparation of the pooled fecal sample and was not reflected on pooled MBRA communities, which looked more similar to other MBRA communities.

The variation between reactor communities and their fecal inocula was more apparent when OTU distributions within specific phyla were examined (Fig. 5b–d); these differences were primarily driven by members of the Firmicutes and Proteobacteria (Fig. 5c, d; also see Additional file 7 which presents Bray-Curtis similarities for MBRA communities and their fecal inocula based upon all OTUs as well as for OTUs partitioned by phyla). The largest diversity across reactors was observed in the Firmicutes; this phylum was represented by 144 OTUs (38 genera from 16 families). Although Proteobacteria were less abundant members of the communities (4–20 % of sequences), they were represented by seven different OTUs across six genera. In contrast, OTUs within the Bacteroidetes phylum were primarily members of the Bacteroides genus (Fig. 5b); all Verrucomicrobia sequences were from a single *Akkermansia* OTU (classified using the Greengenes reference taxonomy as *Akkermansia muciniphila*). From these data, we conclude that stable MBRA communities represent a subset of their fecal inoculum. The percent of the initial fecal inoculum present in core communities vary (from as little as 25 % of sequences (15 % of OTUs) in donor B communities to as much as 49 % of sequences (22 % of OTUs) in donor A communities). The amount of overlap observed was dependent upon the composition of the community present in the initial inoculum and could reflect both differences in abundance of obligate anaerobes that were lost prior to cultivation as well as differences in ability to grow under our cultivation conditions.

### Examination of core OTU dynamics over time in culture

**Fig. 6:**
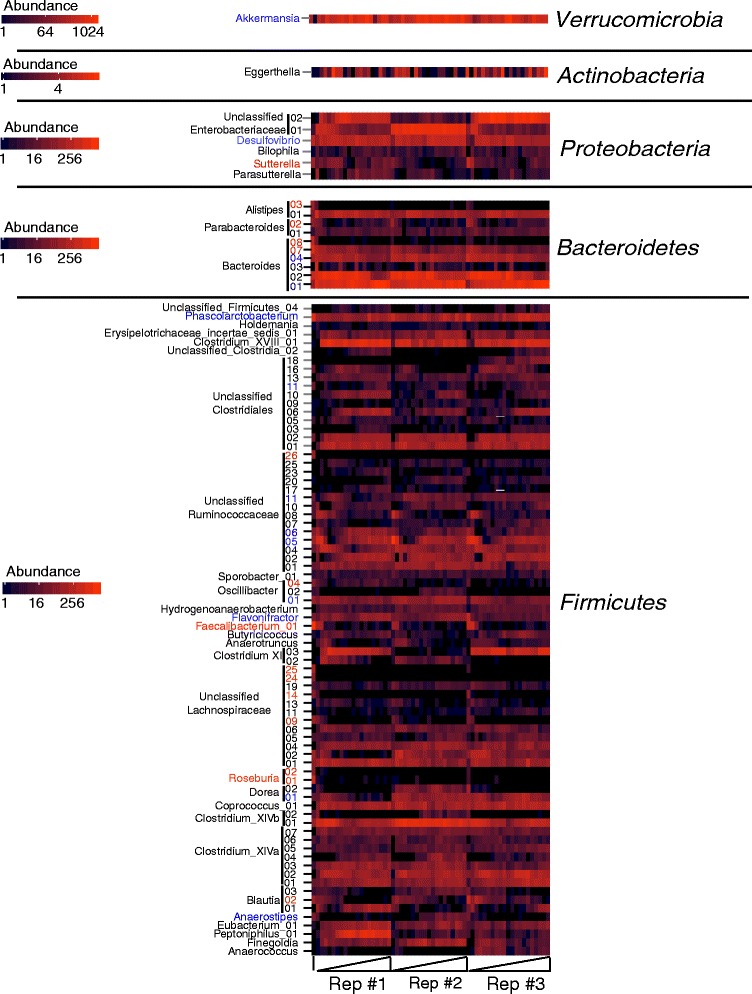
Analysis of abundance of core OTUs identified in Donor A MBRA communities as a function of time in culture. We determined the abundance of the 94 OTUs that were identified as present in individual core communities for MBRAs inoculated with fecal donor A or were abundant in the donor A fecal sample as described in Fig. 5 and plotted the abundance of these OTUs in the fecal sample and over time in culture (days 1–21) across the three replicate reactors. Data are organized by phylum, with the lowest taxonomic classification assigned with confidence listed on the left hand side. Magnitude of shading is indicated on the figure and ranges from 1 to ≥256 sequences for Firmicutes, Bacteroidetes, and Proteobacteria. Abundance of Actinobacteria sequences range from 1 to 4 sequences; whereas the abundance of the single Verrucomicrobia OTU range from 1 to ≥1024 sequences. The line at the left end of the *x*-axis indicates the fecal sample. The *triangles* demarcate time in cultures for the different replicate reactors, with the first time point present on the left side for each replicate. Similar heat maps for donor B and donor C are available in Additional files 8 and 9

Figure 5 presented the average abundance of taxa found in ≥90 % of the samples collected on days 8–21 but did not indicate how levels of these taxa varied over time. To evaluate variation over time, we used heat maps to visualize changes in abundance of core OTUs from the starting fecal samples throughout time in culture (days 1–21). Variation in core OTUs from donor A are shown in Fig. 6, whereas data from donors B and C are shown in Additional files 8 and 9.

Analysis of the data from MBRA communities seeded with donor A demonstrated three general trends. (1) Of OTUs present in the fecal sample, ~20 % were lost or decreased >5-fold during the first several days in culture (e.g., *Faecalibacterium*, *Roseburia*, some Bacteroides, and unclassified Lachnospiraceae; indicated OTUs are identified by red text in Fig. 6). (2) Of OTUs, ~10 % persisted in MBRAs from day-to-day at levels <3-fold different than those observed in fecal samples (e.g., *Flavonifracter*, *Akkermansia*, Bacteroides #4, Ruminococcaceae #5; indicated OTUs are identified by blue text in Fig. 6). (3) The remaining ~70 % of OTUs appear to increase upon cultivation in at least one replicate tested. Similar patterns of increase and loss of OTUs were seen across MBRA communities inoculated from donors B and C and the pooled fecal samples, although the organisms impacted varied across the different fecal donors. For example, *Roseburia* OTUs were lost from donor A and B communities upon cultivation but persisted in communities inoculated with samples from donor C (see Additional file 9, Roseburia OTU is in red type).

Included in the group of OTUs that increase upon cultivation were organisms classified as Enterobacteriaceae, which constituted 2–18 % of total sequences found across replicate reactors (mean abundance of two Enterobacteriaceae OTUs = 8 ± 5 % of sequences). Although many Enterobacteriaceae are facultative organisms, these organisms are unlikely to be respiring aerobically because oxygen levels in the anaerobic chamber are kept below 20 ppm. Organisms from the two Enterobacteriaceae OTUs could be respiring anaerobically, using alternative electron acceptors such as TMAO (known to produced in MBRAs; JMA and RAB, unpublished results) or fumarate (likely metabolic byproduct in reactor), or could be fermenting available carbohydrates.

## Conclusions

In this paper, we demonstrated that MBRAs can be used to efficiently cultivate distinct communities from multiple fecal donors. Within the first week of cultivation, distinct microbial communities capable of metabolizing the available nutrients developed from the different starting inocula. Adaptation to growth in culture shifted the community structure. Although some community members persisted at similar abundances to the fecal inocula, other rare members of the inoculum increased in abundance and a subset of the initial inoculum was lost. This adaptation followed similar trends across replicates, with stable communities obtained by day 8 in culture (7 days with flow; ~21 turnovers).

Day-to-day variation within MBRA communities from single reactors were similar to those observed in stable mouse fecal communities. In contrast, replicate reactors from the same fecal donor exhibited slightly higher variation than was observed between replicate mice. This variation appeared to partly be driven by differing abundances of shared OTUs between replicates and could be indicative of functional redundancy of organisms under the MBRA cultivation conditions. This functional redundancy could allow abundances of OTUs to fluctuate stochastically until stable communities are reached. Further work will be needed to investigate functions of these different communities, although we know that communities formed from all three fecal donors as well as the pooled fecal sample are capable of resisting colonization by *C. difficile* in the absence of perturbation (JMA, CDR, and RAB, unpublished results).

The composition and structure of MBRA communities are similar to those reported in other in vitro models of human fecal communities [27, 28, 39], although the levels of Bacteroidetes are lower than are typically observed in many models. One potential factor that might lead to the lower levels of Bacteroidetes observed under our culture conditions is the low concentrations of fermentable carbohydrates present in our medium, which are a known substrate for Bacteroidetes (reviewed in [40]). However, as the levels of Bacteroidetes detected are similar to those present in the initial fecal inocula, we do not consider the existing levels of Bacteroidetes in MBRA communities to be of concern.

The choice of medium used to cultivate human fecal communities can have a significant impact on the communities that are formed (e.g., [40]). Although many of the existing fecal bioreactor media share some reagents, media can vary significantly in composition from one experimental setup to the next (see Additional file 10, a table comparing media composition from 11 different bioreactor studies). When evaluating media for use in our MBRA model, we compared different published media recipes, with specific emphasis on those models that supported dynamic interactions between human fecal communities and *C. difficile* (i.e., [20]). We also considered the results published by MacFarlane and colleagues [18], which demonstrated significant depletion of fermentable carbohydrates in the third (distal colon mimicking) vessel of their three-stage reactor model. Based upon these observations as well as our own preliminary studies with medium containing higher levels of carbohydrates, we chose to use a medium low in glucose (0.004 %, 220 μM), disaccharides (0.015 % (440 μM) each of maltose and cellobiose), and complex carbohydrates (inulin, 0.02 %; arabinogalactan, 0.01 %), in an attempt to more closely simulate conditions that might be encountered in the distal colon. (Although glucose and maltose are primarily absorbed in the small intestine, inulin, arabinogalacton, and cellobiose have been shown to be primarily fermented in the colon [41–43]). We also included Tween 80, a reagent commonly added to bioreactor medium (see Additional file 10, a table comparing media composition from 11 different bioreactor studies). Tween 80 can be used as a source of unsaturated fatty acids by several *Lactobacillus* species and has been shown to enhance growth [44] and protect from bile acid stress [45].

Although our model does not include a surrogate for mucosal surface as in M-SHIME, our phylogenetic distributions were more similar to the luminal contents of the M-SHIME model than to the original L-SHIME model [27, 39], with Firmicutes composing 44–70 % of the population. Further, we found that sequences of the known mucin-degrader *Akkermansia muciniphila* [46] were present in modest abundance in all stable reactor communities (~5 % of sequences). Further work will be needed to determine how MBRA cultivation conditions are supporting growth of *Akkermansia* in the absence of mucin.

One limitation to the communities cultivated in our MBRA is our inability to cultivate *Faecalibacterium prausnitzii* (11–36 % of sequences from fecal inocula) as well as several other less abundant *Clostridiales* species. Work from the Flint laboratory has demonstrated that both *F. prausnitzii* as well as certain members of *Clostridium* cluster XIVa require acetic acid as a cofactor for metabolism (reviewed in [40]), which is not present in our medium and is unlikely to be produced until a mature community is established. Further, *F. prausnitzii* does not grow well in medium with protein as the primary dietary substrate. Therefore, we may need to consider additional modifications to the medium to include a higher concentration of fermentable carbohydrates as well as a potential source of acetate during outgrowth to facilitate cultivation of *F. prausnitzii.*

A second limitation of our MBRA model is the inability to study interactions with host cells, both to assess how the microbiome impacts the host and to determine feedback of the host upon the microbiome. Although this limitation can be overcome by coupling MBRA studies with follow-up studies in humanized microbiota mice [29], it would also be beneficial to begin to interface MBRA communities directly with host cells in vitro. Platforms for interfacing microbial communities with tissue culture cells have been described [47, 48], including the HMI model for SHIME [47]. We anticipate that the relatively simple MBRA design could make it ideal for coupling with host cells, either individual cell lines or human intestinal enteroids or organoids [49, 50], thereby facilitating higher-throughput in vitro studies of microbiome/host interactions.

## Methods

### Fecal sample collection, preparation, and MBRA operation

Fecal samples from three healthy individuals were collected into sterile containers, sealed, and transferred to an anaerobic chamber within 1 h of defecation. Samples were manually homogenized and subdivided into sterile vials, which were stored at −80 °C until use. Prior to MBRA inoculation, fecal samples were resuspended at 25 % *w*/*v* in anaerobic phosphate buffered saline in the anaerobic chamber, vortexed for 5 min, and centrifuged at 201×*g*. For the pooled sample, equal amounts of each fecal sample (by mass) were combined prior to vortexing.

In order to analyze the impact of freezing upon MBRA cultivation, one fecal donor (donor A) provided a second sample ~3 months post initial donation (donor A2). This sample was collected and transferred to the anaerobic chamber within 1 h of defecation. Following manual homogenization and subdivision into sterile vials, a portion of the sample was flash frozen in liquid nitrogen for 45 min (frozen), whereas the other sample was maintained in the anaerobic chamber until inoculation. Both fresh and frozen samples were then inoculated into triplicate reactors and analyzed as described below. Analysis of this data revealed that there was little impact on communities cultivated from frozen samples compared to freshly voided samples (see Additional file 11 for an NMDS ordination of the Bray-Curtis dissimilarities between these different samples as well tests of community similarity and dispersion).

MBRA were prepared for use as previously described [29] and inoculated with 4 ml of fecal slurry. Bioreactor medium was prepared as described [29], except that 1 g/L of taurocholic acid was replaced with 0.5 g/L of bovine bile, which was added prior to autoclaving. There were multiple reasons for substituting bovine bile for taurocholate. (1) Bovine bile is a complex mixture of bile salts as well as other constituents of bile (e.g., fatty acids, cholesterol, inorganic salts) and is more commonly used in medium for cultivation of human fecal communities than taurocholate alone. (2) Taurocholate was originally included in our medium to promote germination of *C. difficile* spores; subsequent studies have shown that bovine bile is sufficient to support germination under our reactor conditions (Auchtung and Britton, unpublished results). (3) Bovine bile is significantly less expensive than taurocholate (>10-fold lower cost).

After inoculation, fecal bacteria were allowed to equilibrate for 16–18 h prior to the initiation of flow. After equilibration, a 1-ml sample was removed (day 1 sample) and flow commenced at 1.875 ml/h (8-h retention time). Reactors were then sampled daily for 20 additional days (days 2–21). Cells were pelleted from samples by centrifugation at 21,000×*g*. Supernatants were discarded, and pellets were stored at −80 °C until further processed.

### Ethics, consent, and permissions

Fecal sample collection was reviewed and approved by the Institutional Review Board from Michigan State University. All individuals donating samples provided informed consent prior to donation.

### Sample preparation and sequencing

Previously, we had success amplifying *C. difficile* genes from samples that had been disrupted by bead beating without further purification [29]. Further, Flores et al. had also reported success with a direct amplification approach for higher-throughput analysis of 16S rRNA gene content from microbial samples [38]. We were interested in pursuing direct amplification in order to significantly reduce sample preparation time and costs. Therefore, we performed preliminary studies to compare sequences obtained from replicate samples prepared by direct amplification to those that obtained from samples from which DNA was extracted prior to amplification. These studies, which demonstrated robust reproducibility between duplicate samples prepared by direct amplification, are described in detail in Additional file 12: Supplementary Methods and Additional files 13, 14, and 15 (which present the data described in additional file 12).

We resuspended our samples in a 0.5-ml sterile water and transferred them to bead beating tubes. (Our bead beating tubes were prepared by transferring ~200 μl 0.1 mm silica beads (Biospec Products) and 100 μl sterile water into 2-ml screw cap tubes and autoclaving these tubes for at least 20 min prior to use.) Samples were homogenized in a mini-beadbeater-96 (Biospec Products) for 2 min, centrifuged at 8000×g for 1 min, then supernatants were transferred to new tubes, which were stored at -20 °C prior to amplification.

The V4 region of the 16S rRNA gene was amplified with primers F515/R806, using a dual-indexing approach (4 forward primer; 96 reverse primer). The 96-indexed R806 primers used were previously described ([51]; 806rbc0-806rbc96). The indexed F515 primers were essentially as described [37], except that we generated four barcodes that balanced the nucleotide composition at each position (ATCGATGG, TCACGACA, GGTATCTC, and CAGTCGAT) in place of those described by Kozich et al. Prior to PCR amplification, samples were diluted 1:100. The final 25-μl PCR reactions contained 4 μl of diluted template, 1× Phusion High Fidelity Buffer (New England Biolabs), 200 μM dNTPs (Promega), 10 nM primers and 0.225 units of Phusion DNA Polymerase (New England Biolabs). The amplification cycle consisted of an initial denaturation at 98 °C for 30 s, followed by 30 cycles of 10 s at 98 °C, 20 s at 51 °C, and 1 min at 72 °C. Successful amplification was verified by agarose gel electrophoresis of products. If samples failed to amplify, amplification with a new 1:100 dilution was attempted. If re-amplification failed, amplification was attempted with a 1:10 dilution of sample. Because we were unable to obtain amplification from the fecal slurries from donor A, B, C, or the pooled sample at these dilutions, we extracted DNA as previously described [29] prior to amplification and used 4 μl of 10 ng/μl DNA in PCR reactions as described above. As discussed in more detail in Additional file 12, which compares extracted and amplified fecal samples to MBRA communities, comparing MBRA communities that were directly amplified to extracted fecal samples is one potential source of variation between our MBRA communities and fecal samples. However, sample preparation method prior to sequencing is likely not the primary source of variation between fecal and reactor communities as differences in sample preparation method in our control studies resulted in Bray-Curtis similarities of 0.64–0.78 (see Additional file 13 for the impact of sample preparation on Bray-Curtis and Sorenson similarity measures) as compared to the Bray-Curtis similarities observed between fecal and MBRA samples of 0.08–0.40 (mean = 0.17). Further, we were able to successfully amplify community DNA from the donor A2-fresh and frozen samples described in Additional file 11 without DNA extraction, yet observed similar levels of dissimilarity between these fecal inocula and their respective MBRA communities.

Three independent PCR replicates were pooled and cleaned up using AMPure beads as previously described [29]. Concentrations of purified DNA samples were determined with QuantIT (Life Technologies). Purified samples were pooled at equimolar ratios, and the quality of the pooled DNA was assessed by analysis on a Bioanalyzer High Sensitivity DNA Kit (Agilent). Prior to sequencing, DNA concentration was determined by amplicon-specific qPCR (Illumina Complete Kit, Kapa Biosystems). Samples were mixed with 3–7 % phiX DNA, and sequencing was performed at the Research Support Technology Facility (RTSF) with a MiSeq v2 Reagent kit on an Illumina MiSeq running MisSeq Control Software version 2.3.0.3. Sequencing was completed in two separate MiSeq runs.

### Analysis of sequence data

Sequences were analyzed in Mothur versions 1.31, 1.33, and 1.34 essentially as described [37]. MiSEQ SOP version 28 March 2013 was used as a template (http://www.mothur.org/wiki/MiSeq_SOP). Forward and reverse reads were paired, quality-trimmed, aligned to a Silva 16S rRNA gene reference database, trimmed to ensure overlap to the same region of the 16S rRNA gene (position 534-786 of *Escherichia coli* 16S rRNA gene), and pre-clustered to clusters with ≥99 % identity as described. Potential chimeric sequences were identified with the mothur-implementation of uchime and removed. Sequences were then classified with the Bayesian classifier in mothur, using the mothur-formatted ribosomal database project version 9 database from August 2013. Sequences were clustered from a distance matrix using the average-neighbor algorithm in mothur. Taxonomic assignments for each OTU were determined in mothur and are the majority consensus taxonomic assignments for each sequence within the OTU.

The mean and SD of inverse Simpson diversity, Simpson evenness, Shannon diversity, and number of observed species were calculated in mothur from data randomly subsampled to 10,000 sequences over 100 iterations. A single iteration of subsampling to 10,000 sequences was used for determination of Bray-Curtis and Sorenson dissimilarity measures. Bray-Curtis and Sorenson dissimilarity values were calculated on untransformed data, both in mothur and with vegan package of R ([52]; Sorenson = Binary Bray-Curtis); when presented as similarity values, Bray-Curtis and Sorenson similarities = 1-dissimilarities. Non-metric multi-dimensional scaling was also performed in mothur and in vegan using the metaMDS function. NMDS Plots were generated in R from the metaMDS results. ANOSIM, PERMANOVA (ADONIS), and Betadispersion were calculated with the vegan package of R. Heatmaps were generated using the phyloseq implementation of NeatMap [53]. All other plots were generated in Graph Pad Prism v.6.

For the mouse stability studies, paired Illumina reads were downloaded from the Schloss lab website (http://www.mothur.org/MiSeqDevelopmentData.html). Sequences were processed through mothur as described above, then subsampled to 3500 sequences prior to calculation of beta-diversity measures. Data were from three female (F3, F4, and F7) and three male (M2, M5, and M6) mice on days 1–9 (unstable communities) and days 141–150 (stable communities).

## Availability of supporting data

The sequence data described in this manuscript is can be accessed from the short read archive at NCBI (SRP059604).

## Additional files

Additional file 1:
**Additional measures of MBRA diversity.** Plots of Shannon Diversity and the number of OTUs containing at least one sequence. Additional file 2:
**Stabilization of MBRA communities.** Plot of Average Bray-Curtis (BC) dissimilarities on days 8-21 across replicate reactors. Additional file 3:
**Similarity between replicate reactors increases over the first week of cultivation.** Plot of mean BC similarities between replicate reactors over time in cultivation. Additional file 4:
**Mean Bray-Curtis and Sorenson dissimilarities for OTUs in stable and unstable mouse communities.** Table providing mean Bray-Curtis and Sorenson dissimilarities within individual mice and between replicate mice based upon shared OTU content. Additional file 5:
**Relative abundance of all MBRA core OTUs across stable reactors (days 8–21; mean ± SD).** Table providing the mean relative abundance of OTUs present in the core communities of MBRAs of all different fecal types examined. Additional file 6:
**Percent abundance of fecal OTUs absent from stable MBRA core communities.** Table providing the percent abundance of abundant (>0.5 %) fecal OTUs absent from stable MBRA core communities. Additional file 7:
**Bray-Curtis similarities between MBRA communities and their fecal samples determined from OTUs of different phyla.** Table listing Bray-Curtis similarities between replicate MBRA communities of the same fecal type and between replicate MBRA communities and their starting fecal inocula based upon all OTUs as well as by OTUs partitioned by phyla. Additional file 8:
**Analysis of abundance of core OTUs identified in fecal donor B MBRA communities as a function of time in culture.** Heatmap presenting abundance of OTUs that were identified as present in individual core communities for MBRAs inoculated with donor B as well as abundant in the donor B fecal sample. Additional file 9:
**Analysis of abundance of core OTUs identified in fecal donor C MBRA communities as a function of time in culture.** Heatmap presenting abundance of OTUs that were identified as present in individual core communities for MBRAs inoculated with donor C as well as OTUs abundant in the donor C fecal sample. Additional file 10:
**Comparison of reagent concentrations described in 1 L of medium.** Table comparing reagent concentrations described for media used for cultivation of complex fecal communities from 11 different sources. Additional file 11:
**Fresh and frozen fecal samples form similar communities in MBRA.** NMDS plot and ANOSIM, PERMANOVA, and ANOVA of β-dispersion statistics of Bray-Curtis dissimilarities calculated from OTUs present in MBRA communities seeded with donor A2-fresh and donor A2-frozen fecal samples. Additional file 12:
**Supplemental methods.** Supplemental methods describing methods used to compare direct sequencing approach to DNA extraction and amplification as well as identification of potential sequencing contaminants from negative control samples. Additional file 13:
**Impact of sample preparation method on Bray-Curtis and Sorenson similarities in replicate samples.** Table providing differences in Bray-Curtis and Sorenson similarities in replicate samples prepared with different extraction methods. Additional file 14:
**Distribution of sequences in shared OTUs across replicate samples prepared by different methods.** A table providing percent differences in abundance of OTUs and sequences based upon sample preparation method and a graph plotting the abundance of different taxa across replicate samples prepared with different methods. Additional file 15:
**Mean abundance across samples of 25 most abundant OTUs in negative control samples.** Table listing the mean abundance across all samples sequences of 25 most abundant OTUs detected in negative control samples.

## Abbreviations

ANI: average nucleotide identity
BC: Bray-Curtis
MBRA: minibioreactor array
OTU: operational taxonomic unit
SD: standard deviation
