# Supplementary figures and images for: Cultivation of stable, reproducible microbial communities from different fecal donors using minibioreactor arrays (MBRAs)
Source: Microbiome. 2015 Sep 30;3:42. doi: 10.1186/s40168-015-0106-5 (PMC4588258; doi:10.1186/s40168-015-0106-5)

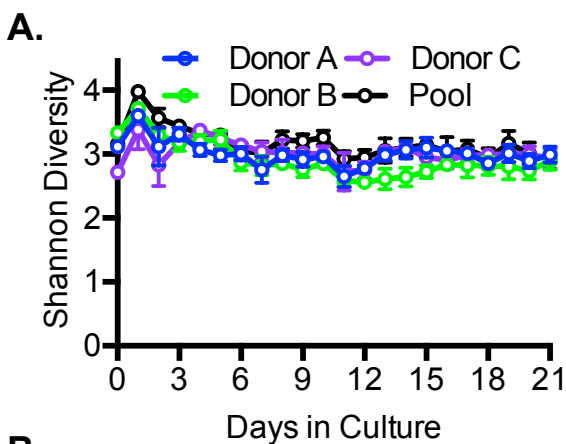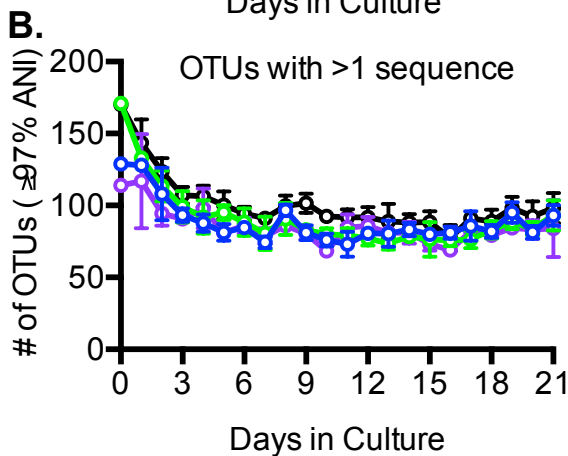

Supplement: Additional file 1: — Additional measures of MBRA diversity. Plots of Shannon Diversity and the number of OTUs containing at least one sequence. [file 40168_2015_106_MOESM1_ESM.pdf]
