# Supplementary material for: Cultivation of stable, reproducible microbial communities from different fecal donors using minibioreactor arrays (MBRAs)
Source: Microbiome. 2015 Sep 30;3:42. doi: 10.1186/s40168-015-0106-5 (PMC4588258; doi:10.1186/s40168-015-0106-5)
Supplement: Additional file 2: — Stabilization of MBRA communities. Plot of Average Bray-Curtis (BC) dissimilarities on days 8-21 across replicate reactors. [file 40168_2015_106_MOESM2_ESM.pdf]

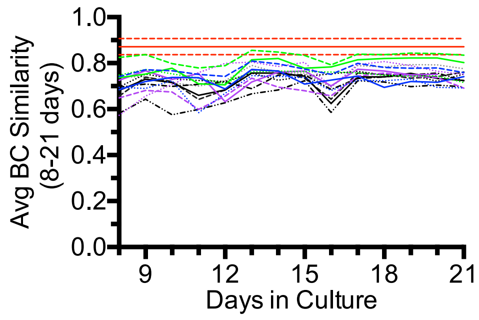

Author
Comment [1]: Figure moved from panel 3B, converted from dissimilarity to similarity instead, and line thickness was reduced to facilitate visualization.

Additional file 2. Stabilization of MBRA communities. Variation in stable communities was assessed by plotting the average Bray-Curtis dissimilarity as in A with communities present in MBRA from days 8-21. The mean Bray-Curtis dissimilarity (red line) ± standard deviation of variation observed in 3 technical replicates is plotted for reference. Donor A – blue lines; Donor B – green lines; Donor C – purple lines; Pool – black lines; Rep 1, solid line; Rep 2, dashed line; Rep 3; dotted line; Rep 4, line with alternating dash and dot; Rep 5, line with alternating dash and two dots; Rep 6, thin solid line.

Author
Deleted: Ind

Author
Deleted: Ind

Author
Deleted: Ind
