# Supplementary material for: Cultivation of stable, reproducible microbial communities from different fecal donors using minibioreactor arrays (MBRAs)
Source: Microbiome. 2015 Sep 30;3:42. doi: 10.1186/s40168-015-0106-5 (PMC4588258; doi:10.1186/s40168-015-0106-5)
Supplement: Additional file 3: — Similarity between replicate reactors increases over the first week of cultivation. Plot of mean BC similarities between replicate reactors over time in cultivation. [file 40168_2015_106_MOESM3_ESM.pdf]

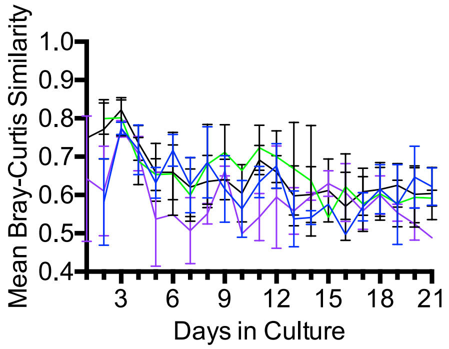

Additional File 3. Similarity between replicate reactors decreases during the first week of cultivation. We determined the mean pairwise BC similarities between replicate reactors and plotted this as a fucntion of time in culture. Blue lines: Donor A; Green lines: Donor B; Purple lines: Donor C; Black: Pool. Error bars represent the standard deviation of the mean.

Author
Deleted: ***<sp>***

Unknown
Formatted: Font:Cambria, Bold

Unknown
Formatted: Font:Cambria

Author
Deleted: ... [1]

Author
Deleted: Diss

Author
Deleted: in

Author
Deleted: over

Author
Deleted: dis

Author
Deleted: Ind

Author
Deleted: Ind

Author
Deleted: Ind
