# Supplementary material for: Cultivation of stable, reproducible microbial communities from different fecal donors using minibioreactor arrays (MBRAs)
Source: Microbiome. 2015 Sep 30;3:42. doi: 10.1186/s40168-015-0106-5 (PMC4588258; doi:10.1186/s40168-015-0106-5)
Supplement: Additional file 4: — Mean Bray-Curtis and Sorenson dissimilarities for OTUs in stable and unstable mouse communities. Table providing mean Bray-Curtis and Sorenson dissimilarities within individual mice and between replicate mice based upon shared OTU content. [file 40168_2015_106_MOESM4_ESM.pdf]

Additional file 4. Mean Bray-Curtis and Sorenson similarities for OTUs in stable and unstable mouse communities.

Author
Comment [1]: Dissimilarities were changed to similarities throughout.

	Within Mouse	Between Mice
**Bray-Curtis**		
Unstable Mouse Community (D0-D09)	0.65 ± 0.11	0.59 ± 0.09
Stable Mouse Community (D141-D150)	0.79 ± 0.06	0.71 ± 0.05
**Sorenson**		
Unstable Mouse Community (D0-D09)	0.74 ± 0.05	0.71 ± 0.05
Stable Mouse Community (D141-D150)	0.76 ± 0.04	0.72 ± 0.04
