# Supplementary material for: Cultivation of stable, reproducible microbial communities from different fecal donors using minibioreactor arrays (MBRAs)
Source: Microbiome. 2015 Sep 30;3:42. doi: 10.1186/s40168-015-0106-5 (PMC4588258; doi:10.1186/s40168-015-0106-5)
Supplement: Additional file 6: — Percent abundance of fecal OTUs absent from stable MBRA core communities. Table providing the percent abundance of abundant (>0.5 %) fecal OTUs absent from stable MBRA core communities. [file 40168_2015_106_MOESM6_ESM.pdf]

Additional file 6: Percent abundance of fecal OTUs absent from stable MBRA core communities

Classification	Donor A	Donor B	Donor C	Pool
Actinobacteria: Bifidobacterium	0.1%	0.1%	0.1%	2.3%
Bacteroidetes: Alistipes	1.5%	0.6%	0.0%	0.1%
Bacteroidetes: Bacteroides	4.5%	0.0%	0.0%	0.0%
Firmicutes: Clostridium_IV	0.4%	3.6%	0.0%	0.7%
Firmicutes: Clostridium_XlVa	0.2%	3.7%	0.2%	0.3%
Firmicutes: Faecalibacterium OTU #1	26.3%	11.5%	35.7%	31.9%
Firmicutes: Faecalibacterium OTU #2	0.0%	2.7%	2.4%	0.5%
Firmicutes: Lachnospiracea_incertae_sedis OTU #1	0.0%	8.8%	7.6%	1.6%
Firmicutes: Lachnospiracea_incertae_sedis OTU #2	0.1%	0.1%	0.0%	0.5%
Firmicutes: Roseburia	0.6%	2.4%	0.5%	0.8%
Firmicutes: Unclassified Clostridia	0.5%	0.0%	0.0%	0.9%
Firmicutes: Unclassified Firmicutes	0.3%	1.0%	0.5%	1.3%
Firmicutes: Unclassified Lachnospiraceae OTU #1	0.1%	4.8%	4.3%	1.4%
Firmicutes: Unclassified Lachnospiraceae OTU #2	0.0%	0.4%	0.2%	2.4%
Firmicutes: Unclassified Lachnospiraceae OTU #3	1.8%	0.8%	0.0%	0.1%
Firmicutes: Unclassified Lachnospiraceae OTU #4	0.5%	0.2%	0.9%	0.7%
Firmicutes: Unclassified Lachnospiraceae OTU #5	0.2%	0.3%	1.2%	0.4%
Firmicutes: Unclassified Lachnospiraceae OTU #6	0.0%	2.0%	0.1%	0.1%
Firmicutes: Unclassified Lachnospiraceae OTU #7	0.0%	0.7%	0.7%	0.6%
Firmicutes: Unclassified Lachnospiraceae OTU #8	0.1%	0.6%	0.0%	0.0%
Firmicutes: Unclassified OTU #1	0.6%	2.5%	1.4%	0.2%
Firmicutes: Unclassified OTU #2	0.0%	0.6%	0.1%	1.0%
Firmicutes: Unclassified Ruminococcaceae OTU #1	0.0%	2.5%	0.0%	0.2%
Firmicutes: Unclassified Ruminococcaceae OTU #2	0.0%	0.3%	0.0%	2.1%
Firmicutes: Unclassified Ruminococcaceae OTU #3	0.9%	0.0%	0.0%	0.0%
Firmicutes: Unclassified Ruminococcaceae OTU #4	0.0%	0.2%	0.0%	0.6%
Unclassified Bacteria OTU #1	0.3%	1.0%	0.7%	3.4%
Unclassified Bacteria OTU #2	0.0%	0.5%	0.0%	1.1%
**Total %**	**39.0%**	**51.7%**	**56.4%**	**55.0%**
