# Supplementary material for: Cultivation of stable, reproducible microbial communities from different fecal donors using minibioreactor arrays (MBRAs)
Source: Microbiome. 2015 Sep 30;3:42. doi: 10.1186/s40168-015-0106-5 (PMC4588258; doi:10.1186/s40168-015-0106-5)
Supplement: Additional file 7: — Bray-Curtis similarities between MBRA communities and their fecal samples determined from OTUs of different phyla. Table listing Bray-Curtis similarities between replicate MBRA communities of the same fecal type and between replicate MBRA communities and their starting fecal inocula based upon all OTUs as well as by OTUs partitioned by phyla. [file 40168_2015_106_MOESM7_ESM.pdf]

Additional file 7. Bray-Curtis dissimilarities between MBRA communities and their fecal samples determined from OTUs of different phyla.

Bray-Curtis similarity between fecal sample and MBRA communities[1]				
	All OTUs	*Firmicutes* OTUs	*Bacteroides* OTUs	*Proteobacteria* OTUs
Donor A	0.2 ± 0.03	0.14 ± 0.03	0.25 ± 0.08	0.28 ± 0.14
Donor B	0.17 ± 0.02	0.04 ± 0.01	0.72 ± 0.07	0.09 ± 0.05
Donor C	0.16 ± 0.02	0.22 ± 0.07	0.29 ± 0.09	0.49 ± 0.23
Pool	0.12 ± 0.01	0.19 ± 0.03	0.33 ± 0.06	0.2 ± 0.05
Sorenson similarity between replicate MBRA communities[2]				
	All OTUs	*Firmicutes* OTUs	*Bacteroides* OTUs	*Proteobacteria* OTUs
Donor A	0.54 ± 0.07	0.5 ± 0.07	0.69 ± 0.15	0.3 ± 0.21
Donor B	0.61 ± 0.08	0.56 ± 0.10	0.75 ± 0.10	0.72 ± 0.14
Donor C	0.55 ± 0.07	0.42 ± 0.06	0.76 ± 0.12	0.51 ± 0.20
Pool	0.57 ± 0.08	0.47 ± 0.10	0.75 ± 0.11	0.57 ± 0.23

[1] BC similarity reported is the mean ± SD between fecal sample and MBRA replicates inoculated with the same donor on Days 8-21.

[2] Sorenson similarity between MBRA communities is the mean ± SD among the pairwise comparisons of replicate MBRA communities inoculated with the same donor on Days 8-21.
