# Supplementary material for: Cultivation of stable, reproducible microbial communities from different fecal donors using minibioreactor arrays (MBRAs)
Source: Microbiome. 2015 Sep 30;3:42. doi: 10.1186/s40168-015-0106-5 (PMC4588258; doi:10.1186/s40168-015-0106-5)
Supplement: Additional file 8: — Analysis of abundance of core OTUs identified in fecal donor B MBRA communities as a function of time in culture. Heatmap presenting abundance of OTUs that were identified as present in individual core communities for MBRAs inoculated with donor B as well as abundant in the donor B fecal sample. [file 40168_2015_106_MOESM8_ESM.pdf]

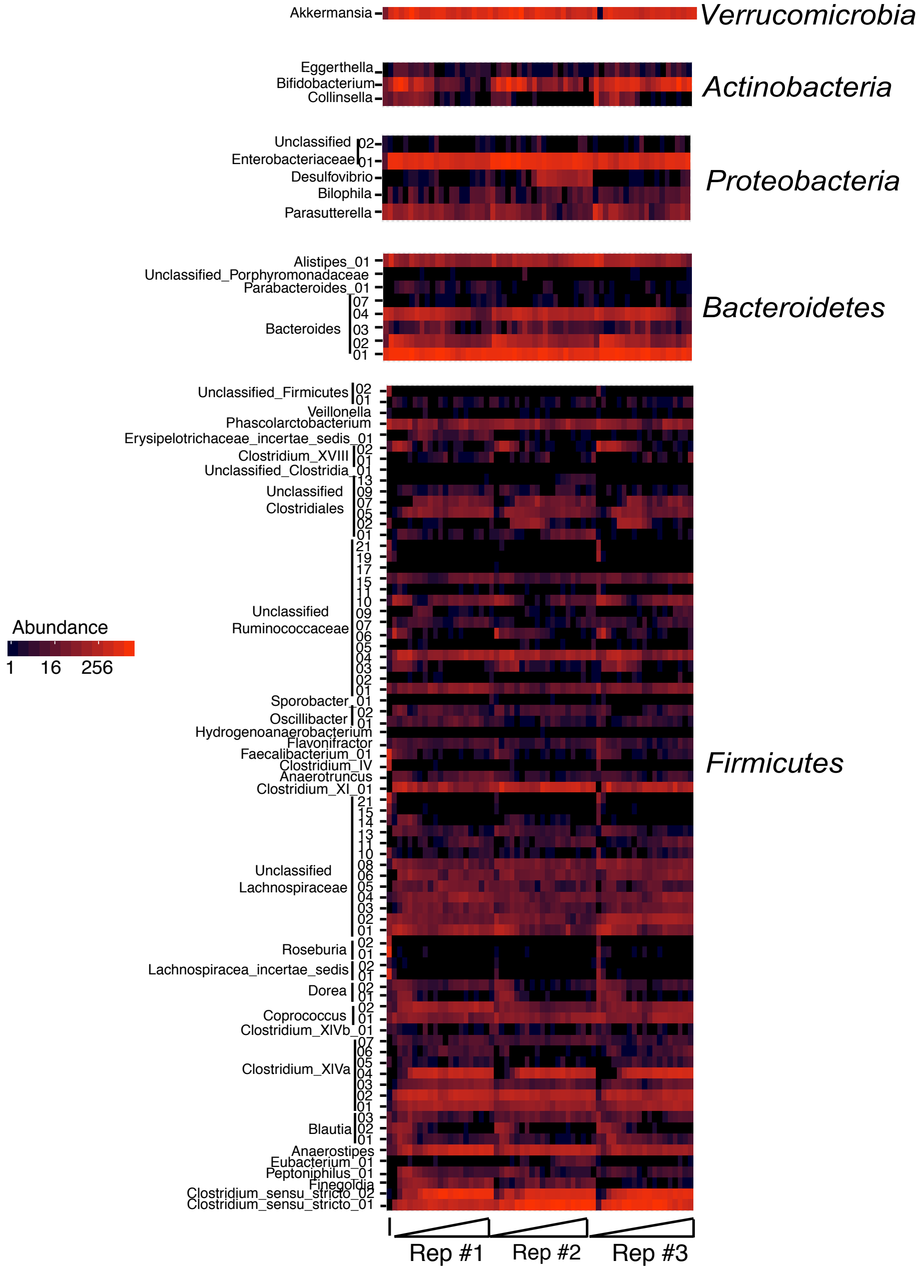

Additional file 8. Analysis of abundance of core OTUs identified in Donor B MBRA communities as a function of time in culture. We determined the abundance of the 93 OTUs that were identified as present in Individual Core

communities for MBRAs inoculated with Donor B or were abundant in the fecal sample from Donor B as described in Figure 5 and plotted the abundance of these OTUs in the fecal sample and over time in culture (Days 1-21) across the three replicate reactors. Data are organized by phylum, with the lowest taxonomic classification assigned with confidence listed on the left hand side. Magnitude of shading is indicated on the figure and ranges from 1 sequence to ≥256 sequences for all phyla. The line at the left end of the x-axis indicates the fecal sample. The triangles demarcate time in cultures for the different replicate reactors, with the first time point present on the left side for each replicate. Similar heat maps for Donor A and Donor C are available in Figure 6 and Additional Files 9, respectively.
