# Supplementary material for: Cultivation of stable, reproducible microbial communities from different fecal donors using minibioreactor arrays (MBRAs)
Source: Microbiome. 2015 Sep 30;3:42. doi: 10.1186/s40168-015-0106-5 (PMC4588258; doi:10.1186/s40168-015-0106-5)
Supplement: Additional file 11: — Fresh and frozen fecal samples form similar communities in MBRA. NMDS plot and ANOSIM, PERMANOVA, and ANOVA of β-dispersion statistics of Bray-Curtis dissimilarities calculated from OTUs present in MBRA communities seeded with donor A2-fresh and donor A2-frozen fecal samples. [file 40168_2015_106_MOESM11_ESM.pdf]

A.

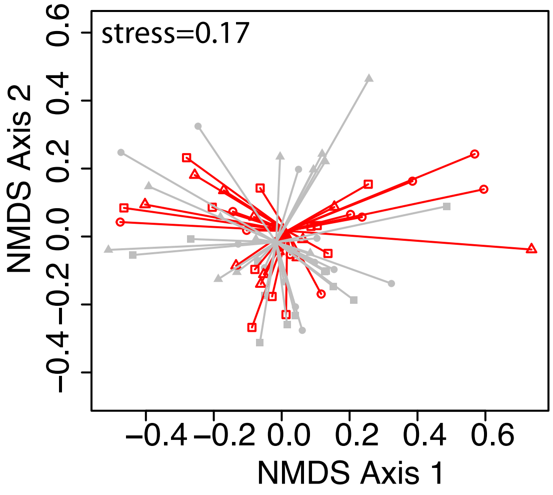

B.

Tests of the differences in inter-sample distances within and between groups:

ANOSIM	R-statistic: 0.05	p-value: 0.01
PERMANOVA	R^2: 0.04	p-value: 0.013

Test for differences in dispersion of samples within group from centroid

ANOVA of β-dispersion	F-stat: 0.0037	p-value: 0.95

Additional file 11. Fresh and frozen fecal samples form similar communities in MBRA. A second fecal sample collected from Donor A was used as a control to monitor the impact of freezing on the ability to cultivate fecal communities. The Donor A2 sample was transferred to the anaerobic chamber and an aliquot was removed, flash frozen and stored at -80°C for 45 min prior to use. Triplicate reactors were inoculated with the fresh sample (maintained in anaerobic chamber ~1 hr) or with the frozen sample and community composition was followed for 14 days as described for the other fecal samples. Bray-Curtis dissimilarities between the fresh and frozen samples on days 2-14 were calculated. In (A) we plotted the Bray-Curtis dissimilarities between samples with NMDS. Gray= Donor A2-Fresh; Red=Donor A2-Frozen; Squares=Replicate 1; Circles=Replicate 2; Triangles=Replicate 3. Lines

connect each replicate time point to the centroid of the group (i.e., IndA2-Fresh or IndA2-Frozen). In (B), we used ANOSIM and PERMANOVA (implemented through the ADONIS function in the vegan package of R) of Bray-Curtis dissimilarities to evaluate the significance of differences in distances between samples within and between groups. Both tests revealed that the differences between the two groups were small, although both tests still had p-values <0.013. The small differences observed did not appear to be a result of differences in variance between the two groups, as ANOVA of the permutated β-dispersion of did not find significant differences in the distance to the centroid. Based upon these results, we conclude that the impact of freezing samples prior to cultivation is small.
