# Supplementary material for: Cultivation of stable, reproducible microbial communities from different fecal donors using minibioreactor arrays (MBRAs)
Source: Microbiome. 2015 Sep 30;3:42. doi: 10.1186/s40168-015-0106-5 (PMC4588258; doi:10.1186/s40168-015-0106-5)
Supplement: Additional file 12: — Supplemental methods. Supplemental methods describing methods used to compare direct sequencing approach to DNA extraction and amplification as well as identification of potential sequencing contaminants from negative control samples. [file 40168_2015_106_MOESM12_ESM.pdf]

**Additional file 12, Supplemental Methods**

Comparison of Sample Preparation Methods

As a comparison of the two sample preparation methodologies, we collected quadruplicate samples from 3 bioreactors on a single day and prepared duplicate samples using the direct amplification approach described in the text and duplicate samples by extraction as previously described [1]. These control reactor communities were from an independent study (Robinson, Auchtung and Britton; unpublished results), using the pooled fecal sample and medium conditions previously described [1]; two reactors (reactors #1 and #2) had been treated with clindamycin and challenged with different strains of *C. difficile*, essentially as described in [1], and the third was not treated with antibiotics and was challenged with *C. difficile,* which had washed out by the time of sample collection. Samples were collected 7 days following challenge with *C. difficile.* (8-days post-cessation of clindamycin-treatment in reactors #1 and #2). We prepared the samples for amplification, sequenced the V4 region of the 16S rRNA gene, and processed the sequence data and binned the data into OTUs with ≥97% ANI as described in the Methods section.

As an initial comparison of the effects of sample extraction method on observed community composition, we determined the Bray-Curtis and Sorenson similarity values between duplicate samples prepared using the same method (See Additional file 13, which presents the impact of sample preparation method on Bray-Curtis and Sorenson similarity measures). We found that duplicate samples prepared by direct

Author
**Deleted:** 4

Author
**Deleted:** un

amplification or DNA extraction and amplification exhibited high levels of similarity (Bray Curtis similarities of 0.89-0.95 for directly amplified samples; Bray-Curtis similarities of 0.88-0.92 for extracted and amplified samples). Similarity measures based upon OTU presence/absence decreased with both methods, although this decrease was larger for samples prepared by DNA extraction and sequencing compared to directly amplified samples (Sorenson similarities of 0.76-0.78 for directly amplified samples; Sorenson similarities of 0.56-0.61 for extracted and amplified samples). From these data, we conclude that both sample preparation methods result in similar communities. However, samples that were extracted prior to amplification appear to have higher numbers of low abundance OTUs that are not shared between replicates compared to directly amplified samples.

We also compared replicate samples prepared with different sample preparation methods. We observed 1.2-1.4 decreases in Bray-Curtis similarities compared samples prepared with the same method (mean Bray-Curtis similarities of 0.64 ± 0.03 - 0.78 ± 0.04 across different reactor communities; See Additional file 13, which presents the impact of sample preparation method on Bray-Curtis and Sorenson similarity measures). Sorenson similarities also decreased 1.2-1.3-fold in samples prepared by different methods compared to samples prepared by direct amplification. From these data, we conclude that sample preparation method impacts measures of community composition based upon both OTU abundance and presence/absence.

To better evaluate how preparation methods impacted OTU distribution in replicate samples, we examined the distribution of OTUs and sequences that were shared across replicates prepared with the two different methods. We found that although only 45-55% of total OTUs were shared across replicates, these OTUs contained 97-99% of the total sequences. (See Additional file 14 panel A, which presents the percent of total OTUs and sequences from each replicate that were contributed by OTUs shared across all four replicates).

We also plotted the distribution of sequences found in OTUs shared across all four replicates organized by taxonomic classification at the family level. (See Additional file 14 panel B). Sequence abundance varied by less than 2-fold across sample preparation method for 14 of the 22 families we examined. For the remaining eight families, only two exhibited consistent differences across all three bioreactor communities. Sequences classified in the family *Erysipelotrichaceae* were detected at 2-5-fold higher levels in directly amplified samples compared to extracted and amplified samples. (The mean abundance in directly amplified samples was 5.1% ± 2.5%; whereas, the mean abundance in extracted and amplified samples was 1.3% ± 0.6%. Evaluation of these differences in abundance with a two-tailed student's t-test corrected for unequal variances resulted in a p-value=0.012). Sequences classified in the family *Ruminococcaceae* were detected at 2-3-fold higher levels in directly amplified samples compared to extracted and amplified samples. (The mean abundance in directly amplified samples was 4.5% ± 2.0%; whereas, the mean abundance in extracted and amplified samples was 1.9% ± 1.1%. Evaluation of

these differences in abundance with a two-tailed student's t-test corrected for unequal variances resulted in a  p-value=0.025).  Although there were decreases in abundance detected in directly amplified samples compared to extracted in amplified samples, these differences were not consistent across different reactor communities.

From these data, we conclude that direct amplification is a feasible approach for comparison of MBRA community samples because of its robust reproducibility across replicates as well as its decreased sample preparation costs. Although most of the sequences were found in shared OTUs across different sample preparation methods, differences in sample preparation did result in differences in measures of community similarity. Therefore, these differences should be considered when comparing samples prepared with different methods.

Identification of potential contaminating OTUs within sample data

In order to assess the impact of experimental sources of contamination on community composition, we also sequenced eight negative control samples. These samples were processed along with all other experimental samples and analyzed as described in the methods to generate OTUs with ≥97% identity. From our OTU table randomly subsampled to 10,000 sequences, we calculated the average abundance and rank order of each OTU across samples of each type (Negative Control, n=8; Extraction Control, n=12; Donor A, n= 60; Donor B, n=61; Donor C, n=62; Pool, n=122; Donor A, Sample 2 – Fresh (Donor A2 - Fresh), n=39 and Donor A2 – Frozen, n=40).  We then sorted these OTUs by rank abundance in negative control samples,

Author
**Deleted:** When we compared the communities from samples prepared using the two different methods, we found that the community composition between replicate samples were similar, although there was more variation between replicate samples prepared with different methods than between methods. (see Additional file 15). In spite of these differences, replicate samples prepared using the two different methods were more similar to each other than those replicate samples from other reactors (see Additional file 15).

Author
**Deleted:** Individual

Author
**Deleted:** Individual

Author
**Deleted:** Individual

Author
**Deleted:** Individual

Author
**Deleted:** Ind

Author
**Deleted:** Ind

selecting the top 25 OTUs for further analyses. The 25 most abundant Negative Control OTUs contained a mean of 78.1 ± 5.7% (SD) of each of the eight negative controls' sequences. Looking at the sequence distribution of these OTUs across the samples, we found six OTUs that were abundant in negative control samples and were present at low abundance in all other samples (see Additional file 15, OTUs designated by #). We suspect that sequences from these OTUs represent contaminants introduced by our sample preparation and extraction methods. Therefore, these OTUs were excluded from further analyses.

In addition, we also found OTUs that were abundant in both negative control and experimental samples (OTU abundances marked by asterisks in Additional file 15 were found in the top 25 most abundant OTUs in the indicated sample types). We suspect that the abundance of these OTU's sequences in the negative control samples are due to cross-contamination with low levels of template from neighboring samples on the 96-well plate during the initial 16S rRNA gene amplification step. Because the abundance of these OTUs vary across sample types, we think it is unlikely that the sequences from these OTUs have been detected as a result of contamination from other sources, although we cannot completely exclude this possibility. The potential for environmental contamination in low biomass samples was recently discussed in detail [2] and there is not yet a consensus for how to deal with sequences detected in negative controls (e.g., [3,4]).

**Supplemental References**

1. Robinson CD, Auchtung JM, Collins J, Britton RA. Epidemic Clostridium difficile Strains Demonstrate Increased Competitive Fitness Compared to Nonepidemic

Isolates. Infect and Immun. 2014; 82:2815–25.

2. Salter SJ, Cox MJ, Turek EM, Calus ST, Cookson WO, Moffatt MF, et al. Reagent and laboratory contamination can critically impact sequence-based microbiome analyses. BMC Biol. 2014; 12:87.

3. Flores GE, Caporaso JG, Henley JB, Rideout JR, Domogala D, Chase J, et al. Temporal variability is a personalized feature of the human microbiome. Genome Biol. 2014; 15:531.

4. Goodrich JK, Di Rienzi SC, Poole AC, Koren O, Walters WA, Caporaso JG, et al. Conducting a Microbiome Study. Cell. 2014;158; 250–62.

Author
**Deleted:** ion

Author
**Deleted:** ity

Author
**Deleted:** Jun 17

Author
**Deleted:** (7)

Author
**Deleted:** Nov 5

Author
**Deleted:** 1–12

Author
**Deleted:** Flores GE, Caporaso JG, Henley JB, Rideout JR, Domogala D, Chase J, et al. Temporal variability is a personalized feature of the human microbiome. 2014 Dec 3;:1–13.

Author
**Deleted:** Elsevier Inc;

Author
**Deleted:** Jul 17

Author
**Deleted:** (2)
