# Supplementary material for: Cultivation of stable, reproducible microbial communities from different fecal donors using minibioreactor arrays (MBRAs)
Source: Microbiome. 2015 Sep 30;3:42. doi: 10.1186/s40168-015-0106-5 (PMC4588258; doi:10.1186/s40168-015-0106-5)
Supplement: Additional file 13: — Impact of sample preparation method on Bray-Curtis and Sorenson similarities in replicate samples. Table providing differences in Bray-Curtis and Sorenson similarities in replicate samples prepared with different extraction methods. [file 40168_2015_106_MOESM13_ESM.pdf]

Additional file 13. Impact of sample preparation method on Bray-Curtis and Sorenson similarity measures.[1]

**Impact of sample preparation method on measures of community similarity between replicate samples from the same reactor**			
	Reactor #1	Reactor #2	Reactor #3
**Bray-Curtis**			
Similarity between duplicate samples prepared by direct amplification	0.95	0.93	0.89
Similarity between duplicate samples prepared by extraction and amplification	0.92	0.88	0.88
Similarity between replicate samples prepared by different methods[2]	0.72 ± 0.03	0.78 ± 0.04	0.64 ± 0.03
**Sorenson**			
Similarity between duplicate samples prepared by direct amplification	0.76	0.77	0.78
Similarity between duplicate samples prepared by extraction and amplification	0.59	0.61	0.56
Similarity between replicate samples prepared by different methods[2]	0.58 ± 0.02	0.58 ± 0.05	0.62 ± 0.02

[1]Sequences partitioned into OTUs with ≥97% ANI (V4 region of the 16S rRNA gene)
[2]Mean and SD of 4 pairwise-similarity measures.
