# Supplementary material for: Cultivation of stable, reproducible microbial communities from different fecal donors using minibioreactor arrays (MBRAs)
Source: Microbiome. 2015 Sep 30;3:42. doi: 10.1186/s40168-015-0106-5 (PMC4588258; doi:10.1186/s40168-015-0106-5)
Supplement: Additional file 14: — Distribution of sequences in shared OTUs across replicate samples prepared by different methods. A table providing percent differences in abundance of OTUs and sequences based upon sample preparation method and a graph plotting the abundance of different taxa across replicate samples prepared with different methods. [file 40168_2015_106_MOESM14_ESM.pdf]

Additional File 14. Distribution of sequences in shared OTUs across replicate samples prepared by different methods

A.

	% of OTUs shared across replicates prepared by different methods[1]	% of sequences found in OTUs shared across replicates prepared by different methods[1]
Reactor #1: Direct Amplification	48.6% ± 4.6%	98.5% ± 0.1%
Reactor #1: Extraction and Amplification	44.6% ± 4.6%	99% ± 0.3%
Reactor #2: Direct Amplification	54.6% ± 5%	98.9% ± 0.1%
Reactor #2: Extraction and Amplification	44.5% ± 3.9%	98.7% ± 0.1%
Reactor #3: Direct Amplification	50.5% ± 2.3%	97.2% ± 0.4%
Reactor #3: Extraction and Amplification	49.5% ± 7.1%	99.1% ± 0.3%

[1]Data presented are the mean ± SD of technical duplicates.

B.

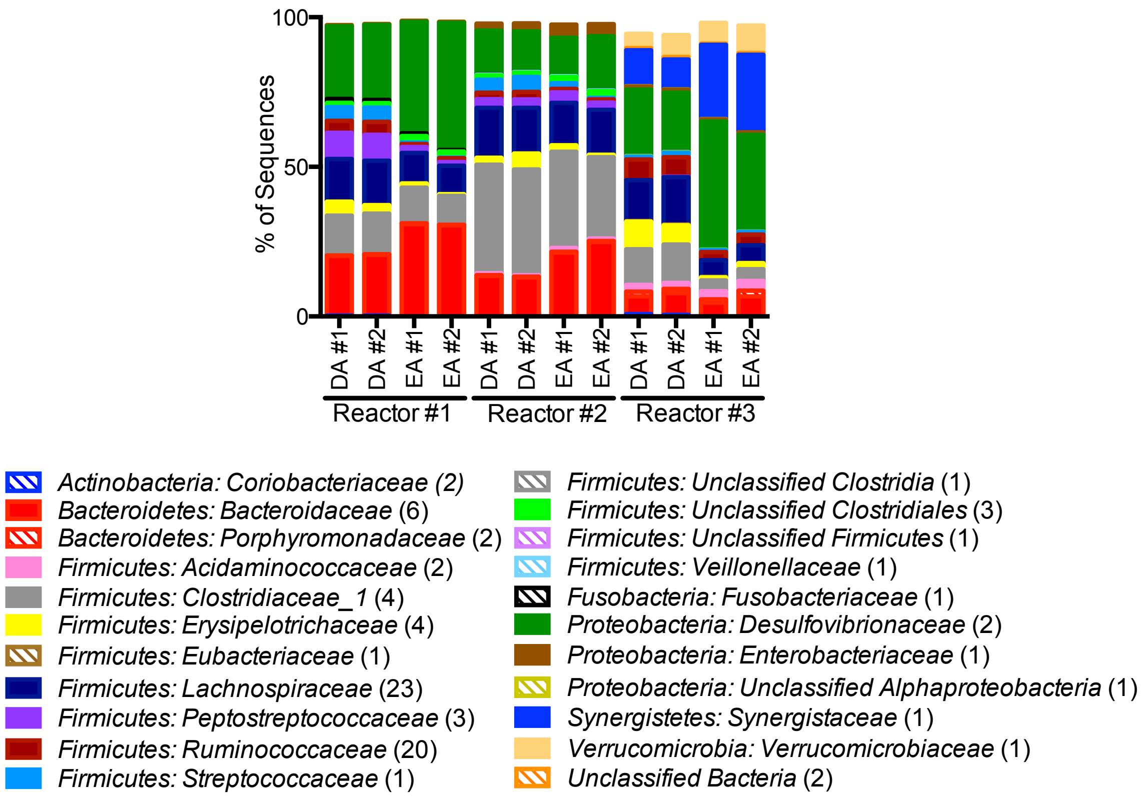

In panel A, we calculated the percent of total OTUs and sequences from each technical replicate that were contributed by OTUs shared across all four technical replicates (both sample preparation methods). We present the mean ± the standard deviation of the technical duplicates prepared by the same method for each of the three reactor communities. In panel B, we plot the distribution of sequences found in OTUs shared across technical replicates (as described in A) organized by taxonomy. To better facilitate visualization, data presented combines all OTUs from the same family into a single value. The phylum and family labels are presented, except when taxonomic resolution at the family level could not be assigned with confidence. In those cases, the lowest taxonomic level that could be assigned with confidence is presented. The numbers in parentheses indicate the total number of OTUs represented by each family.
