# Supplementary material for: Cultivation of stable, reproducible microbial communities from different fecal donors using minibioreactor arrays (MBRAs)
Source: Microbiome. 2015 Sep 30;3:42. doi: 10.1186/s40168-015-0106-5 (PMC4588258; doi:10.1186/s40168-015-0106-5)
Supplement: Additional file 15: — Mean abundance across samples of 25 most abundant OTUs in negative control samples. Table listing the mean abundance across all samples sequences of 25 most abundant OTUs detected in negative control samples. [file 40168_2015_106_MOESM15_ESM.pdf]

Additional file 15. Mean % abundance across samples of 25 most abundant OTUs in negative control samples

Rank	OTU Name	Lowest Taxonomic Classification	Negative Control	Method Control	Donor A	Donor B	Donor C	Donor A2 - Fresh	Donor A2 - Frozen	Pool
1	Otu0002	Bacteroides	14.2 ± 5.5	6.2 ± 4.4*	11.5 ± 8.2*	2.1 ± 2.8*	0.2 ± 0.4	16 ± 6.7*	16.9 ± 7.6*	3.7 ± 4*
2	Otu0059	Halomonas#	13.1 ± 7.2	0.2 ± 0.1	0.2 ± 0.2	0.2 ± 0.3	0.2 ± 0.2	0.4 ± 0.2	0.6 ± 0.8	0.2 ± 0.1
3	Otu0001	Bacteroides	12.4 ± 8.5	4.5 ± 3.7*	14.5 ± 8.1*	14.3 ± 4.2*	5.4 ± 3.2*	10.4 ± 3.7*	11.3 ± 4.7*	16.2 ± 6.2*
4	Otu0034	Bilophila	5 ± 2.1	25.3 ± 10.8*	0.1 ± 0.1	0.1 ± 0.1	0.1 ± 0.1	0.1 ± 0.1	0.2 ± 0.4	0.1 ± 0.1
5	Otu0100	Shewanella#	4.8 ± 2.5	0.1 ± 0	0.1 ± 0.1	0.1 ± 0.1	0.1 ± 0.1	0.1 ± 0.1	0.2 ± 0.3	0.1 ± 0.1
6	Otu0040	Oscillibacter#	3.9 ± 1.8	0.5 ± 0.3	0.1 ± 0.2	0.5 ± 0.5	0.1 ± 0.1	0.1 ± 0.1	0.2 ± 0.3	0.6 ± 0.4
7	Otu0004	Enterobacteriaceae	3.2 ± 1.7	1.5 ± 1.5*	5.8 ± 7.1*	7.7 ± 3.7*	3.8 ± 2.9*	7.2 ± 4.1*	5.8 ± 3.5*	6 ± 3.1*
8	Otu0035	Peptoniphilus	2 ± 2.5	0	2.5 ± 4.4*	0.2 ± 0.3	3.2 ± 3.4*	1.1 ± 0.8*	0.9 ± 1.2	0.7 ± 1.7
9	Otu0011	Clostridium_XVIII	1.9 ± 2.4	0	6.3 ± 3.8*	0.1 ± 0.1	1.5 ± 1.5*	3.6 ± 2.5*	4 ± 2.3*	4.4 ± 5.4*
10	Otu0069	Bacteroides#	1.5 ± 0.7	0	0	0	0	0	0.1 ± 0.1	0
11	Otu0047	Clostridium_XlVb#	1.5 ± 0.8	0	0	0.6 ± 0.7	0	0	0.1 ± 0.1	0
12	Otu0086	Sutterella#	1.4 ± 0.7	0.6 ± 0.6	0	0	0	0	0.1 ± 0.1	0
13	Otu0013	Alistipes	1.3 ± 0.7	0	2.2 ± 1.5*	1.6 ± 1.7*	4.5 ± 3.2*	1.7 ± 1.3*	1.7 ± 1.1*	4.7 ± 3*
14	Otu0026	Enterobacteriaceae	1.3 ± 1.9	0.1	7.3 ± 8.4*	0	0.1 ± 0.2	0.3 ± 0.3	0.6 ± 1	2.3 ± 4.6*
15	Otu0044	Firmicutes	1.3 ± 0.6	0.1 ± 0	0.1 ± 0.2	0	1.4 ± 2*	0	0.1 ± 0.1	0.8 ± 1.4
16	Otu0007	Bacteroides	1.2 ± 0.6	2.2 ± 2.2*	0.3 ± 1.1	0.1 ± 0.2	20.4 ± 9.1*	0	0.1 ± 0.1	0.7 ± 1.5
17	Otu0012	Clostridium_XI	1.2 ± 0.7	1.1 ± 0.7*	0.1	3.3 ± 2.9*	1 ± 1.1	0	1.1 ± 2.4*	1.1 ± 1.3*
18	Otu0018	Lachnospiraceae	1.1 ± 0.6	0.1 ± 0.1	1.3 ± 1*	1 ± 1.2*	1.5 ± 1.5*	3 ± 1.9*	2.8 ± 1.3*	0.6 ± 0.6
19	Otu0010	Clostridium_XlVa	1 ± 0.4	4.7 ± 4.4*	1.8 ± 1.3*	3.1 ± 1.5*	1.2 ± 0.8*	1.3 ± 1.2*	0.8 ± 0.5	2.2 ± 1.3*
20	Otu0027	Desulfovibrio	1 ± 0.9	0.3 ± 0.2	2.9 ± 1.3*	0.5 ± 0.7	0.1	3.2 ± 1*	3.4 ± 0.9*	2.2 ± 1.1*
21	Otu0098	Acidaminococcaceae	1 ± 0.6	1.2 ± 1.4*	0	0	0	0	0.1 ± 0.1	0
22	Otu0008	Clostridium_XlVa	0.9 ± 0.6	0.3 ± 0.4	1.1 ± 0.8*	1.3 ± 0.7*	1.3 ± 0.7*	0.8 ± 0.5	0.7 ± 0.4	1.4 ± 1.3*
23	Otu0025	Ruminococcaceae	0.9 ± 0.2	0.3 ± 0.2	0.8 ± 0.9	1.6 ± 1.3*	0	1.6 ± 1*	1.6 ± 1*	1.1 ± 1*
24	Otu0032	Phascolarctobacterium	0.9 ± 0.8	0.4 ± 0.5	2 ± 2.2*	0.8 ± 0.7	0.1	2.4 ± 1.5*	2.1 ± 0.9*	1.5 ± 1.6*
25	Otu0016	Clostridium_XlVa	0.8 ± 0.3	2.8 ± 3.2*	0.6 ± 0.5	0.2 ± 0.2	0.3 ± 0.2	0.4 ± 0.2	0.7 ± 0.3	0.8 ± 1.5

* Indicates Mean OTU abundances in the top 25 most abundant OTUs for the indicated sample.

# Indicates those OTUs found in abundance only in negative control samples.
